# Research Tracks in Orthopedic Surgery: Analyzing Participation, Outcomes, and Academic Contribution

**DOI:** 10.7759/cureus.113895

**Published:** 2026-08-03

**Authors:** Leah C Quinn, Michelle R Shimizu, Ava Jennings, Shreya M Saraf, Mary K Mulcahey

**Affiliations:** 1 Stritch School of Medicine, Loyola University Chicago, Maywood, USA; 2 Orthopedic Surgery, Loyola University Medical Center, Maywood, USA

**Keywords:** dedicated research years, orthopaedic surgery research, orthopaedic surgery research track, research during residency, research years

## Abstract

Background: Research is an important component of orthopedic surgery residency programs. Dedicated research years during residency are increasingly popular among surgical specialties, including orthopedic surgery. The purpose of this study was to characterize the prevalence, timing, and institutional characteristics of dedicated research years within orthopedic surgery residency programs and to assess career outcomes associated with completing a research year.

Methodology: Two hundred and twelve orthopedic surgery residency programs were identified using the Fellowship and Residency Electronic Interactive Database (FREIDA). Program websites were reviewed to extract information regarding research tracks and participating residents. The Hirsch index (H-index) and total publications were recorded using the Scopus database (Elsevier) to measure research output.

Results: Thirty-one (14.6%) orthopedic surgery residency programs offered a research year for their residents; most (23/31, 74.2%) were categorized as university-based. The most popular time to take a research year was after the second year of residency (12/23, 52.2%). Research-track alumni (RR, *n *= 50) demonstrated significantly higher H-indices (8.1 vs. 6.0, *P *= 0.008) and total publications (23.1 vs 16.1, *P *= 0.009) than non-research residents (NRR, *n *= 126). Among alumni with known current practice type (*n *= 95), RR graduates were more frequently in academic practice (59.3% vs. 38.2%), although this difference did not reach statistical significance (*P *= 0.071). Male residents demonstrated a higher RR participation rate than female residents (75/277, 27.1% vs. 18/90, 20.0%), although this difference was not statistically significant (*P *= 0.18).

Conclusions: Dedicated research years continue to emerge within orthopedic surgery residency, appearing most commonly at university-based institutions. Participation in a structured research track may support continued scholarly productivity, though its long-term influence on career trajectory remains unclear. Transparent information about research tracks and alumni could facilitate further evaluation of the impact of a dedicated research year in orthopedic surgery training.

## Introduction

Participating in research is an important component of orthopedic surgery residency programs in the United States [1]. The importance of research is reflected by the increasing emphasis on research productivity in the selection process for U.S. orthopedic surgery residency positions, particularly among top-tier research output programs [2]. As of 2024, the National Resident Matching Program reported that matched U.S. orthopedic surgery residency applicants had a mean of 23.8 research products, up from 14.3 in 2020 [3,4]. Involvement with research during residency may influence a resident’s career trajectory [5,6]. As of 2020, only 45% of orthopedic surgery residency programs reported providing protected research time, and program directors reported a 50.8% satisfaction level with the resident research experience [7]. The same year, the Accreditation Council for Graduate Medical Education (ACGME) began requiring orthopedic surgery residency programs in the United States to provide residents with a minimum of 60 days of protected research time [8]. This was an addition to the existing ACGME requirement that each resident must participate in and publish or present their research [9]. One strategy programs may use to meet this requirement while fulfilling all clinical requirements is to offer a dedicated research year.

The benefits of protected research time remain debated. It is unclear whether protected research time during residency improves research output. A 2021 study by Voss et al. investigated the effects of dedicated research time in German-speaking orthopedic surgery residency programs in Austria, Germany, and Switzerland and found that protected and planned research time yielded more self-reported research activity measured in hours per week among residents [10]. Another study by Krueger et al. found the opposite [11]. The authors analyzed three U.S. military-based orthopedic surgery residency programs with differing dedicated research time requirements (research year required for all residents, one volunteer research resident per year, no dedicated research time). They found no difference in the quantity or quality of research publications among residents. Other studies have demonstrated that structure is key to improving research productivity during protected research periods [12,13].

Offering dedicated research years during residency training has become increasingly popular among surgical specialties. As of 2023, 41% of U.S. general surgery residency programs offered a dedicated research year to their residents [14]. Greater transparency on how different U.S. orthopedic surgery residency programs incorporate the ACGME research requirements and the prevalence of programs that support dedicated research years may better allow prospective residents to make informed decisions regarding residency selection. Additionally, examining how residency programs incorporate and structure dedicated research years may provide insight for programs seeking to enhance their research productivity and academic contributions. The purpose of this study was to characterize the prevalence, timing, and institutional characteristics of dedicated research years within orthopedic surgery residency programs, and to assess career outcomes associated with completing a research year, including research productivity, academic practice, and fellowship subspecialty choice.

## Materials and methods

Data included in the final analysis were collected between November and December 2025. This study did not require IRB approval, given the nature of the work involving the review of publicly accessible websites. 

Research track residency programs 

Two hundred and twelve orthopedic surgery residency programs were identified using the Fellowship and Residency Electronic Interactive Database (FREIDA) and confirmed for 2025-2026 accreditation using the ACGME website [15,16]. Further information was identified through (1) the program website links provided by FREIDA or (2) institution websites found by Google search. All programs with an accessible website and current resident webpage were included in this study; programs without were excluded.

The variables collected for each program were determined based on previous literature. Institution name, location, webpage, and institution type were collected from FREIDA. Institutional websites were reviewed independently by two authors for information on program structure and availability of protected research opportunities. For programs offering protected research tracks (one or more years of dedicated research time), additional data were collected on track length, number of track residents, availability of advanced degrees, and research track alumni.

Resident data 

Resident data including name, research productivity, and gender were extracted from 19 program websites, Scopus, and NPIdb.org. Only residents from programs who publicly listed current, past, or future research-track participants were included. Research-track residents (RR) were compared to a non-research resident cohort (NRR) selected from the same program and graduating year to match the RR alumni by graduation year and institution to ensure that RR outcomes were compared to peers from the same environment and time period. This was done to minimize confounding variables due to graduation year effects. Residents were further divided into two analytic cohorts for comparison with matched peers: (1) current RRs who have not yet completed their research year and their matched NRR peers (*n *= 186); and (2) RR alumni who were at least two years post-research year and their matched NRR peers (*n* = 176). Residents who did not fit into either cohort were excluded from research productivity analysis.

Research productivity was measured using Scopus-derived total publication count and Hirsch index (H-index) [17]. Scopus author profiles were verified using resident name, residency institution, graduation year, fellowship institution, current practice location, institutional affiliation, and publication topics when available. When multiple Scopus profiles were identified for the same individual, profiles were reviewed for consistency with available institutional and publication history. Residents with ambiguous or unverifiable Scopus profiles were excluded from analyses.

Metrics were collected for RR and RR alumni and their NRR peers for comparison. Additional data collected included gender data (obtained via NPIdb.org), fellowship subspecialty, and current practice setting. Current practice setting was categorized as academic, private, or mixed. Academic practice was defined as employment at an institution with an affiliated orthopedic surgery residency program, medical school, or university-based faculty appointment. Private practice was defined as employment in a non-academic private group or hospital-based practice without an identified academic appointment. Mixed practice was defined as employment involving both private or community-based clinical practice and an identifiable academic affiliation, teaching role, or faculty appointment.

Statistical analyses were conducted using Python to summarize the data. Group comparisons between RR and NRR were performed to evaluate differences in research productivity and career outcomes. Independent-samples Welch’s t-tests were used to compare mean H-indices and total publication counts between groups. Comparisons of current practice setting (academic, private, or mixed) were restricted to alumni with publicly available practice information and were evaluated using chi-square or Fisher’s exact tests as appropriate. Fellowship choice distributions between RR and NRR were also compared using chi-square tests. Additional comparisons were made across graduation year and gender. Residents with missing data for a specific analysis were excluded from that analysis, resulting in slightly different denominators across outcomes. Statistical significance was defined as *P *< 0.05.

## Results

Research track residency programs 

Of the 212 orthopedic surgery residency programs reviewed, 31 (14.6%) offered a research year. Most of the 31 programs were university-based (23/31, 74.2%), followed by community-based university-affiliated (5/31, 16.1%), and other types, including military-based, community-based, or uncategorized (3/31, 3.2% each). Most programs (17/31, 54.8%) offered one research track position per class, six (19.4%) programs offered two, and the remainder offered four (1/31, 3.2%), six (1/31, 3.2%), or unspecified positions (6/31, 19.4%).

Selection for the research year varied: 17/31 (54.8%) programs matched residents separately into their research track using ERAS, three (9.7%) required internal applications during the first or second year of residency, and seven programs (22.6%) mentioned residents could choose to participate in a research year. One program (3.2%) required an application to the research program before residency unrelated to the ERAS application system, and one (3.2%) required all residents to participate in the research year. Three programs (9.7%) did not specify the method of resident selection. Among 29 programs that specified the length of the research track, most (28/29; 96.6%) offered one-year research programs. The most popular timing to take a research year was between the second and third year of residency (12/23, 52.2%), but variation was noted among the 23 programs that specified timing. Three (9.7%) programs offered associated advanced degrees (MPH, Master of Research, or PhD).

Current RR

Of 31 programs with research track options, 17 (54.8%) listed current research track residents who had not yet completed their research year. A total of 186 current orthopedic surgery residents with research track designation were included in the analysis, comprising 42 RR and 144 NRR. The mean H-index was slightly higher among RR compared with NRR (4.4 ± 4.0 vs. 3.9 ± 3.4), although this difference was not statistically significant (Welch’s t-test, *P *= 0.453). Similarly, mean total publication counts were higher among RR compared to NRR residents (15.24 ± 21.1 vs. 11.12 ± 11.4); however, this difference also did not reach statistical significance (*P* = 0.230). When stratified by postgraduate year (PGY), no statistically significant difference in H-index or total publications was observed between RR and NRR residents at any PGY level (all *P *> 0.15).

Overall, while RR tended to demonstrate higher average research output across PGY levels, variability and modest RR sample sizes limited statistical significance.

RR alumni

Across the 176 alumni identified from 11 programs reporting RR graduates, 50 completed a research track (RR), and 126 did not (NRR). RR alumni had significantly higher mean H-Indices (8.1 ± 4.2 vs. 6.0 ± 5.8; mean difference 2.1; 95% confidence interval (CI), 0.56-3.69; *P* = 0.008) and total publication counts (23.1 ± 15.2 vs. 16.1 ± 17.6; mean difference 7.0; 95% CI, 1.75-12.28; *P* = 0.009) compared to their NRR counterparts.

**Figure 1:**
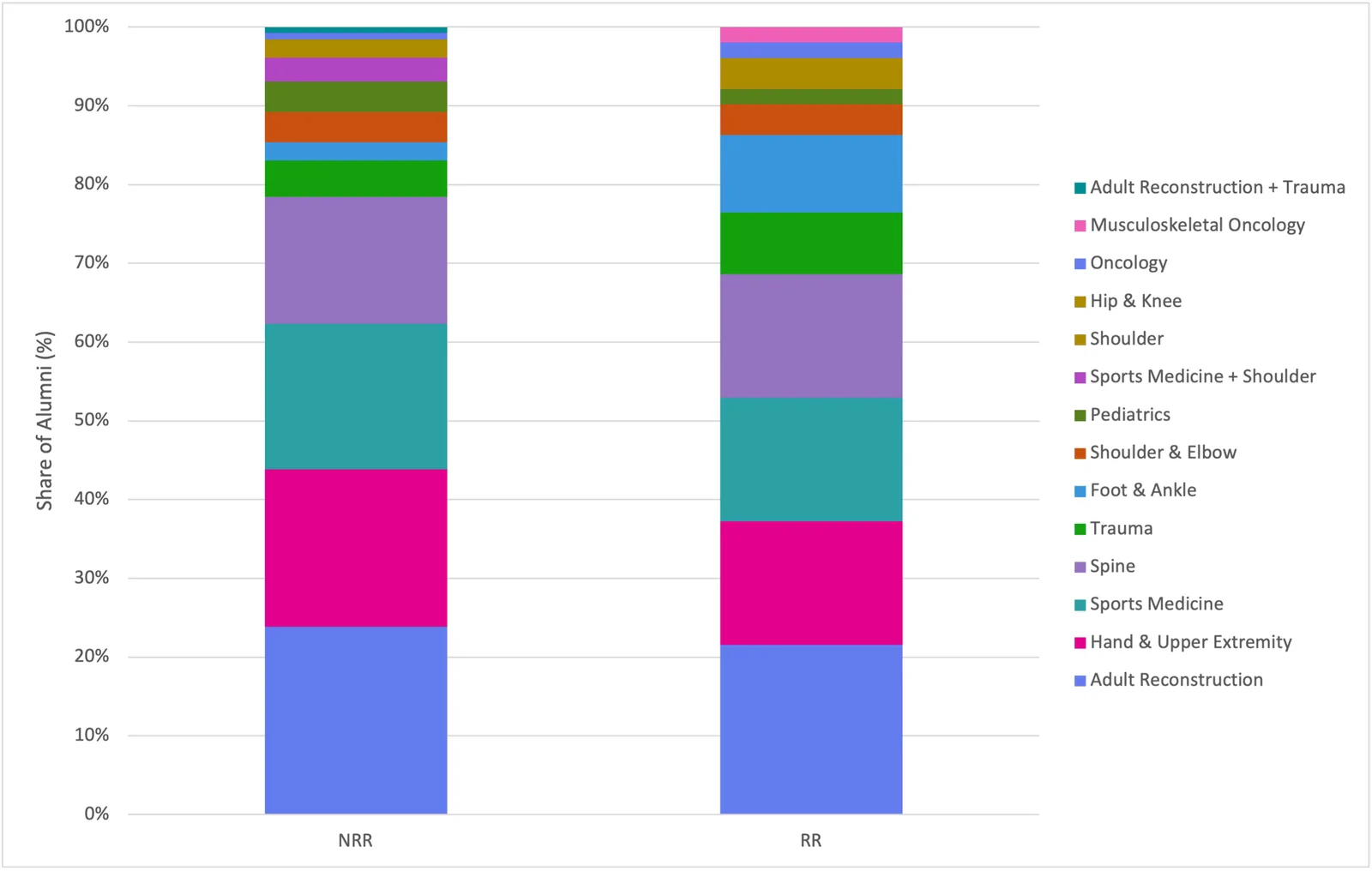
Distribution of graduated orthopedic surgery residents by subspecialty choice among research residents (RR) and non-research residents (NRR). Stacked bar charts illustrate the proportional representation of subspecialty selections for each cohort. Image generated by the authors using Microsoft Excel for Mac (Version 16.104; Microsoft Corporation, Redmond, WA).

There were no significant differences in fellowship subspecialty choice between RR and NRR graduates (chi-square test of independence; χ²=14.31, degrees of freedom (df) = 13; *P* = 0.35). Both groups pursued Adult Reconstruction, Hand/Upper Extremity, Sports Medicine, and Spine fellowships in similar proportions (Figure 1). 

**Figure 2:**
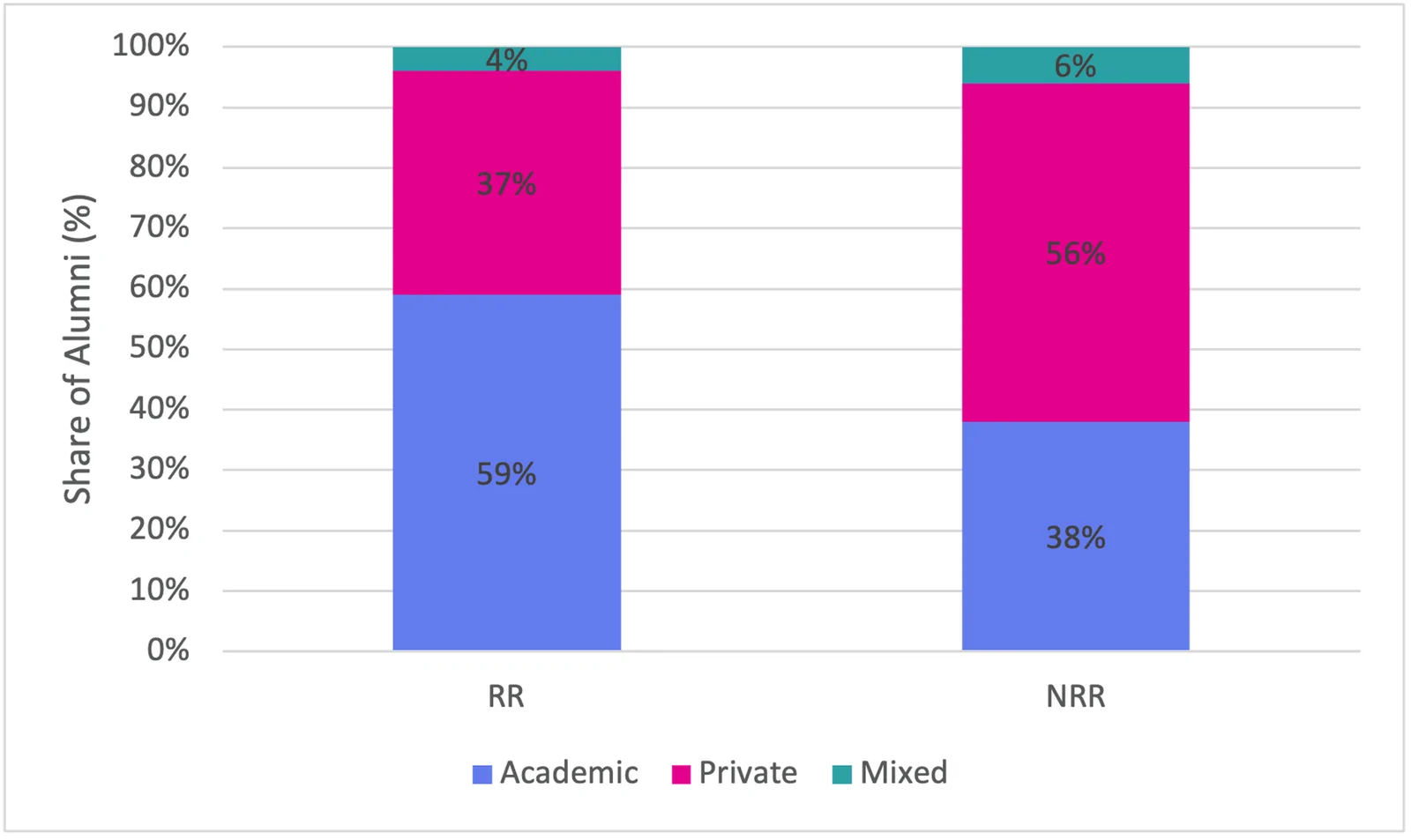
Distribution of graduated orthopedic surgery residents by current practice type among research residents (RR) and non-research residents (NRR). Stacked bar charts display the proportion of graduates practicing in academic, private, or mixed practice settings for each cohort. Percentages represent the relative distribution within each group. Image generated by the authors using Microsoft Excel for Mac (Version 16.104; Microsoft Corporation, Redmond, WA).

Of 95 graduates with known current employment locations, practice patterns showed a non-significant trend towards more RR graduates pursuing academic careers (RR 16/27, 59.3% vs. NRR 26/68, 38.2%; 95% CI, -0.8% to 42.9%; Fisher’s exact two-sided *P* = 0.071) (Figure 2, Table 1). RR alumni were more likely than NRR alumni to return to their residency training institution, although this difference did not reach statistical significance (RR 7/27, 26.0% vs. NRR 10/68, 14.7%, 95% CI, -7.3% to 29.8%; Fisher’s exact (two-sided) *P* = 0.24).

**Table 1:** Distribution of graduated orthopedic surgery residents across current practice type. Academic versus non-academic practice types were compared between RR and NRR graduates using Fisher’s exact two-sided test (*P* = 0.071).

Practice type	RR (*n* = 27), *n* (%)	NRR (*n *= 68), *n* (%)
Academic	16 (59.3%)	26 (38.2%)
Private	10 (37.0%)	38 (55.9%)
Mixed	1 (3.7%)	4 (5.9%)

Gender-based comparisons 

A total of 367 residents with available gender and research output data were analyzed. Overall, 277 residents (75%) were male and 90 (25%) were female. Within the RR cohort, 75 residents (81%) were male and 18 (19%) were female. Among male residents, 75 of 277 participated in a research track (27.1%), compared with 18 of 90 female residents (20.0%). Male residents demonstrated a higher RR participation rate than female residents, although this difference was not statistically significant (two-proportion testing *P* = 0.18, 95% CI, -2.7% to 16.9%).

## Discussion

This multi-institution, cross-sectional study aimed to characterize the prevalence, timing, and institutional characteristics of dedicated research years in U.S. orthopedic surgery residency programs, and to assess career outcomes associated with completing a research year. In total, 31 of 212 (14.6%) orthopedic surgery residency programs offer residents one or more research years, most commonly a single year (28/29; 96.6%) between the second and third years of residency (12/23; 52.2%). Only three (9.7%) programs mentioned the opportunity to pursue an advanced degree.

Programs and structure 

Programs offering a structured research track were disproportionately university-based (23/31, 74.2%) compared to the overall proportion of FREIDA-listed university-affiliated orthopedic residency (103/212, 48.6%) programs. This suggests that access to structured research tracks may be limited in community or military-based programs, consistent with prior work noting resource limitations in non-academic settings [7]. Notably, 25 of 31 (80.6%) programs offering a research year were listed within the top 50 research productivity orthopedic surgery residency programs according to a publication quantity and quality productivity ranking as shown by Jones et al., and 21 of 31 (67.7%) were among the top 50 by reputation according to Doximity [18,19]. Previous studies have identified fewer orthopedic surgery programs offering dedicated research years; Mittwede et al. [6] reported 23 programs, and Barghi et al. [7] found seven. This discrepancy may reflect different methods of obtaining data (survey vs web-based) or an increase in programs offering or advertising research years after the implementation of the 2020 ACGME requirement to provide 60 days of protected research time [8]. As of 2020, most orthopedic surgery residency programs required research for graduation, but only 45% reported providing protected research time [7]. The change in ACGME requirements may have prompted more programs to offer a dedicated research year.

The majority of orthopedic surgery residency research years were shorter in length (1 year) than the mean length reported in general surgery programs (1.7 years) [20]. The popular timing for research years between the second and third year of residency may allow residents to build a solid foundation of clinical knowledge early while providing sufficient time to complete projects before applying for fellowships. General surgery residency programs were similarly found to provide dedicated years after the second or third year of residency [14]. Early placement of research years during residency may help mitigate declines in learned surgical skills, although the optimal timing of research years remains poorly studied [21]. Future studies should explore the effects of research year timing on clinical skill development.

Interestingly, the only orthopedic surgery residency program found to require a mandatory research year was military-based. The institution website highlighted the mandatory research year as a reason their residents match favorably into fellowship programs [22]. This differs notably from general surgery, where 41% of general surgery residency programs offer, and 8% require at least one research year [14]. Because research years are better established in general surgery, most research on dedicated research years is limited to general surgery. Our study suggests that orthopedic surgery is trending toward broader adoption of research years, thereby warranting further exploration.

Residents and alumni outcomes 

Among 176 alumni from 11 programs (2017-2026 graduates), RR alumni (RR, *n* = 50) had significantly higher mean H-indices (8.1 vs. 6.0, *P* = 0.008) and total publications (23.1 vs. 16.1, *P* = 0.009) than NRR (*n* = 126). The H-index accounts for both the number of publications and the number of citations each publication receives, providing a measure of both productivity and impact. Higher H-indices among RR alumni suggest greater research volume and impact within the orthopedic literature.

Notably, among residents who had not yet completed a dedicated research year, no statistically significant differences in H-index or total publication counts were observed between RR and NRR. This suggests that baseline research productivity was comparable between groups before completion of the research year. In contrast, statistically significant differences in both H-index and total publication counts emerged among RR alumni compared to their matched peers, despite similar proportions across pre- and post- research year analyses. While the analysis of current residents may be underpowered due to limited sample size, this pattern raises the possibility that participation in a structured research year contributes to subsequent differences in research productivity.

Future studies should explore program and resident motivations for incorporating dedicated research years, including whether research tracks are intended to enhance academic productivity, support fellowship placement, or allow residents flexibility to pursue research interests.

Current practice type analysis (*n* = 95) revealed that a substantial percentage of RR alums (16/27, 59.3%) compared to NRR alums (26/68, 38.2%) are currently affiliated with academic institutions, although this correlation did not reach statistical significance (*P* = 0.071). A similar non-significant trend was noted for RR alumni returning to their residency institution (7/27, 26.0%; *P* = 0.24). These trends could indicate the potential influence of structured research programs on career trajectories toward academic orthopedic surgery. Future studies will benefit from a larger sample size to further elucidate the correlation.

Previous single-institution studies reported mixed effects of research years on academic career trajectory and research productivity [5,23-25]. Smaller samples may have limited the ability to detect differences in post-residency outcomes. Our multi-institution, cross-sectional study provides broader evidence that RR participation is associated with increased research output and a trend toward academic practice, supporting the idea that structured research opportunities support academic engagement. 

Limitations 

This study utilized publicly available program and resident data, which may be incomplete or inconsistent. The information available on program websites varies significantly. Because RR status was determined from publicly available program websites, programs with dedicated research opportunities that were not advertised online may have been misclassified as not offering a research track. Additionally, programs that publicly advertise research tracks or list RR participants may have more formalized or robust research infrastructure than programs that do not, potentially introducing selection bias. The availability of public information regarding research opportunities and residents depends on each institution's discretion. Many residency program websites did not include the names of alums who participated in the research track, making it difficult to identify their fellowships, current institutions, and titles. Therefore, the information we extracted may not be generalizable to all orthopedic surgery residency programs across the United States.

Second, to ensure a homogeneous final number of publications for each resident, only publications indexed in Scopus were included in this study. This excludes all publications from poster presentations and non-indexed work. There is likely a discrepancy between residents' true number of research products and that which is reflected in this study.

Additionally, analysis did not adjust for program type or prior research experience. It was not possible to determine which research products were produced during residency versus before matriculation or after graduation, limiting the ability to attribute research output specifically to residency experiences.

While our results suggest that RR have higher H-indices and publication counts, the current dataset (limited to 27 RR alumni with complete training, including 16 who graduated before 2020) does not allow us to fully evaluate how much of this difference persists beyond residency. A longitudinal analysis would ideally examine research productivity post-graduation to determine the lasting impact of a research year. This limitation reflects the fact that many residency programs do not publicly list past RR participants, restricting the available data. Increased transparency on program websites would enable further understanding of the outcomes of RR alumni.

Finally, gender was identified using publicly available databases (NPIdb), which may lead to misclassification. Administrative data may not reflect self-identified gender.

Given the inherent limitations associated with the study's design, future studies may benefit from nationwide or global survey distribution, further investigating the topic of research tracks and opportunities in orthopedic surgery residency programs.

## Conclusions

Dedicated research years continue to emerge within orthopedic surgery residency, appearing most commonly at university-based institutions. Participation in a structured research track may support continued scholarly productivity, though its long-term influence on career trajectory remains unclear. Transparent information about research tracks and alumni could facilitate further evaluation of the impact of a dedicated research year in orthopedic surgery training.
